# Strengthening the role of hospital leadership in infection control (LEAD-IC) – a multimodal educational intervention in German acute care hospitals

**DOI:** 10.1186/s12909-023-04709-z

**Published:** 2023-10-11

**Authors:** Sonja Hansen, Cornelius Remschmidt, Christin Schröder, Michael Behnke, Petra Gastmeier

**Affiliations:** 1 Charité – Universitätsmedizin Berlin, corporate member of Freie Universität Berlin, Humboldt-Universität Zu Berlin, and Berlin Institute of Health, Institute of Hygiene and Environmental Medicine, Berlin, Germany; 2 National Reference Centre for Surveillance of Nosocomial Infections, Berlin, Germany

**Keywords:** Infection prevention and control, Training and education, Leadership, Hospital management, Patient safety

## Abstract

**Background:**

The effect of leadership support for adherence to infection control and prevention (IPC) measures has been demonstrated. To expand this support, a target group-specific educational study for chief medical officers (CMO) was implemented and its influence on IPC indicators was investigated.

**Methods:**

A controlled cohort study was conducted between 2018 and 2019. The intervention based on an initial workshop, an e-learning course, and a final meeting. Participants’ activities involving IPC management were surveyed. Consumption of alcohol hand rub (AHR) and incidence density of hospital-associated (HA) *Clostridioides difficile*-associated infections (CDI) were analyzed.

**Results:**

Eight percent of 360 CMOs invited participated in the initial workshop; 70% of those participants registered for the online course. Overall, 43% completed the post-intervention questionnaire, in which 85% of respondents reported increased collaboration with relevant stakeholders. The pre-intervention median AHR consumption was higher in the intervention group than in the control group. Both groups showed an increase (38.6 (interquartile range (IQR) 33.6; 45.0) to 41.9 ml/patient day (PD) (IQR 35.0; 56.6) and 33.4 (IQR 28.3; 40.8) to 35.8 ml/ PD (IQR 31.6; 43.2), respectively). Pre-intervention median HA CDI cases were lower in the intervention group than in the control group. Both groups reported a decrease (0.22 (IQR 0.17; 0.33) to 0.19 cases/1000 PD (IQR 0.15; 0.26) and 0.32 (IQR 0.2; 0.48) to 0.22 cases/1000 PD (IQR 0.11; 0.33), respectively).

**Conclusion:**

Multimodal IPC training of CMOs is worthwhile and can lead to changes in IPC-relevant cooperation in hospitals. IPC training of hospital management should be further intensified.

**Supplementary Information:**

The online version contains supplementary material available at 10.1186/s12909-023-04709-z.

## Introduction

Infection prevention and control (IPC) is essential for patient safety in healthcare. Recent data on healthcare-associated infections (HAIs) in German acute care hospitals show that around 5% of patients suffer from HAIs. This corresponds to an estimated 400,000- 600,000 HAIs per year [1, 2]. Of those, at least one-third can be prevented by enhanced implementation of IPC measures [3–5]. The global increase in antimicrobial resistance (AMR) emphasizes even more the need to reduce transmission of pathogens and hence of infections. It implies the need for hospitals to intensify their IPC measures [4].

Several key components have been identified as crucial to organizing and carrying out IPC programs.. These include organizational structures, safety culture, and engaged leadership [5–8]. In addition to supervision of routine processes, management can impact the capacity to foster change and innovation in healthcare regardless of their professional group or level of leadership [7, 9–11].

Hospital leadership has been shown concretely to have a positive impact on IPC in health care workers’ (HCW) attitudes, knowledge, and practices for the prevention of Methicillin-resistant *Staphylococcus aureus* (MRSA) and Carbapenem-resistant *Enterobacteriaceae* [12, 13].

In addition to supporting HCWs’ adherence to IPC recommendations, hospital-based leadership may help hospital-based IPC teams, for example, in the context of IPC campaigns [14]. According to Brannigan et al., IPC should be a general priority at all levels and, rather than being the responsibility of a single individual or a small dedicated team, and should be integrated in all management systems [15]. Already decades ago, Goldmann et al. specified strategic goals and actions as the challenge to hospital leadership in combatting the problem of AMR [16].

Training and education have always played an important role in improving IPC. They generate awareness, including awareness of the theoretical background of measures. However, training and education in IPC and AMR have thus far been aimed primarily at HCWs and IPC professionals rather than other occupational groups.

In order to expand important IPC knowledge and to empower managers at the hospital level in their (formal) roles in IPC, a program was developed and offered to German chief medical officers (CMOs).

The aims of this study were (i) to strengthen the competence of hospital management regarding IPC-measures through a multimodal training program in order (ii) to improve IPC management at the hospital level. Because we are not aware of any similar study approach, the feasibility of the project was assessed as well.

## Methods

### Study design and course of the study

An interventional, controlled cohort study was conducted between 2018 and 2019 as part of the German national surveillance system KISS [17]. Eligible participants were CMOs of acute hospitals where surveillance of i.) alcohol-based handrub use in the module “HAND-KISS” and of ii.) *Clostridioides (C.) difficile*-associated diarrhoea in the module “CDAD-KISS” had been conducted for at least two years [18, 19]. Non-acute care hospitals were not considered.

Invitations were sent to the CMOs of all eligible hospitals in which the purpose and course of the study was described. Those who agreed to participate were invited to an initial one-day workshop (kick-off) meeting in Berlin, Germany in January 2018.

Participants and their respective hospitals were included in the analysis as an intervention group if they agreed to participate, gave written informed consent, and took part in the kick-of meeting. Eligible, non-participating hospitals were defined as the control group.

### Multimodal IPC training program

The multimodal IPC training program consisted of (i) the kick-off meeting followed by (ii) a 6-week e-learning training course. The project was completed by (iii) another one-day face-to-face meeting one year after the kick-off meeting. All parts of the IPC-training program were developed by authors of the study (CR, PG & SH) using national (e.g. German Commission for Hospital Hygiene and Infectious Disease Prevention [20]) and international IPC recommendations and guidelines [7, 21]. The content of all training items was adapted to the respective role of participants, e.g. organizational aspects were given priority over practical aspects of IPC.

(i) At the initial one-day workshop, basic IPC principles, such as IPC structure and interdisciplinarity, implementation, legal requirements, outbreak management, and the role of antibiotic stewardship in IPC, were presented and discussed with the participants. In addition, before the start of the intervention, participants’ opinions regarding their role in IPC were collected and summarized as quotes.
(ii) The e-learning course was developed in cooperation with the German Hasso Plattner Institut in Potsdam (“openHPI”). OpenHP is an educational internet platform that offers knowledge by means of learning videos, tutorials, and homework [22]. The 6 week-e-learning course consisted of 12 learning videos (video length 5 to 15 min), in which the authors of the study (CR, PG & SH) presented the following IPC-topics:
  1. Core components of IPC in hospitals
  2. Surveillance strategies in healthcare facilities
  3. Multimodal strategies for implementing IPC activities
  4. Organization of IPC education and training in hospitals
  5. Relevance of screening for resistant bacteria in hospital settings
  6. Management of nosocomial outbreaks

All participants were required to perform an initial online registration. Registered participants were sent weekly email reminders to watch newly released IPC-videos. Although viewing videos in chronological order was recommended by the authors, it was also possible to watch videos in any order at one's own discretion. All videos were available online from 03/2018 to 05/2018 and were accessible only to participants of the study. It was possible to download the presentation slides used in each video as well as to download the presentation slides from the kick-off meeting. Participation rates in the course were monitored with the openHPI software. Homework or self-tests were not required for completing the online course.

(iii) At the closing event, preliminary results and participants’ experiences were discussed.

### Endpoints

Primary endpoints were changes in IPC management during the intervention stated in a questionnaire, which was sent to participants before the closing event. The structured questionnaire consisted of five questions regarding possible changes in the participants’ interaction with (i) members of the hospital management team (e.g. chief executive officer (CEO), head physicians) or (ii) with the IPC team itself. (iii) Whether there were changes in the organization of regular IPC-meetings; (iv) whether the hospitals’ internal IPC-guidelines had been revised and (v) whether specific IPC measures (e.g. antibiotic stewardship) had been implemented. Response options were yes/no along with the option to provide additional information.

Secondary endpoints were the collection of hospital-based data on (i) the total AHR consumption and (ii) the incidence of *Clostridioides* (C.) *difficile*-associated infections (CDI) -cases in the year before, during, and after the intervention (2017–2019). For this purpose, data were analysed on the basis of general KISS methods [17]. In brief, AHR consumption, CDI and patient days (PD) are monitored by the hospitals and submitted yearly to the German National Reference Center for Surveillance of Nosocomial Infections at Charité Universitätsmedizin – Berlin.

### Statistical analysis

We used descriptive statistics to compare baseline characteristics of the intervention group with the control group. Regarding primary endpoints, we descriptively analyzed changes in IPC organization or management in the intervention group. For each question, we expressed data as n of N in percentage.

In evaluating secondary endpoints, PD and total AHR consumption per year were used to calculate the median and the interquartile range of AHR consumption per PD (ml/PD) on the hospital level. Data on CDI cases were expressed as total CDI prevalence (total CDI-cases/100 patients) and as hospital-associated CDI incidence density (hospital-associated CDI-cases/1000 PD). CDI cases were considered hospital-associated if symptoms first occurred after 3rd day of admission.

An analysis of secondary endpoints was carried out (i) for all hospitals in the intervention and control groups and (ii) for a defined "core group" of the intervention and control groups, in which only hospitals that recorded data continuously during 2017–2019 in the national surveillance system KISS were taken into account.

Differences between the cohorts were tested with the chi-square test. The change in the secondary endpoints were analyzed in the core group and the differences between 2017 and 2019 were tested with the paired Wilcoxon rank sums test. Analyses were carried out using R 3.4.3 [R Core Team (2013); R Foundation for statistical computing, Vienna, Austria].

### Ethical considerations, data protection, and study registration

Participation was voluntary and only possible after a consent form had been submitted. The study was approved by the institutional review board of Charité-Universitätsmedizin Berlin (EA4/161/17). The authors declare that written informed consent was obtained from all individual participants included in the study. All methods were carried out in accordance with relevant guidelines and regulations or the ‘Declaration of Helsinki’. An institutional data protection officer assured adherence with data protection laws. The study was registered at the German Clinical Trials Register (DRKS00013016) on 22/09/2017. Initially, the study was designed as a randomized interventional study in a stepped-wedge design. Since the response rate of eligible participants was lower than expected and we were not able to achieve our calculated sample size, we decided, following the recruitment process, to change the study design to a prospective cohort study.

## Results

Thirty-three (9.7%) of 360 eligible CMOs from 11 of 16 German federal states agreed to participate. Due to short notice cancellations, 30 CMOs (8.3%) participated in the end and took part in the kick-off meeting. Three hospitals sent a deputy CMO. Only one participant was female.

The median number of beds per hospital was significantly lower in the intervention group than in the control group (300 (interquartile range (IQR): 178; 493) and 357, IQR: 25; 595, respectively) (*p* = 0. 04). In terms of the level of care hospitals in the intervention group had a statistically significant higher level of medical care compared to the control group (Supplement Table 1).

### Participation in the e-learning course

Twenty-one (70%) of the participants registered for the online training course, of which 17 (56%) watched more than 50% of the teaching videos and 8 (27%) completed the course. The presentation slides from the initial meeting were downloaded by all registered participants. Thirteen (43%) of the CMOs participated in the closing session of the project.

### Primary endpoints

Overall, 13 of 30 participants completed the post-intervention questionnaire. Eleven of the 13 respondents stated that they had changed the way they collaborate with head physicians, nurse managers, or local public health agencies. In particular, IPC issues were discussed more frequently with the various professional groups. With regard to local public health agencies, participants stated that options for better cooperation were discussed. Five respondents stated that collaboration with the hospital’s IPC team and the organization of IPC commission meetings had been improved by an increase in the number of regular meetings and by more intensive discussions of relevant IPC issues prior to IPC commission meetings (Table 1).

**Table 1:** Changes in IPC management during the intervention as reported by participants (*n* = 13), LEAD-IC Study

Aspect of change	n/N	%
Intensified communication with		
-Head physicians	11/13	85
-Hospital administration	9/13	69
-Nurse managers	8/13	62
-Chief executive officer	6/13	46
-Local health authorities	5/13	38
-IPC team	5/13	38
Change in hospital IPC guidelines	8/13	62
More intense organization of IPC commission meetings	5/13	38
Initiation and implementation of IPC measures	13/13	100

*IPC* Infection prevention and control

Regarding changes in internal IPC-guidelines, 8 (62%) of the respondents said that they had either revised their hospital’s internal IPC-manual or optimized its operation procedures for outbreaks. All participants stated that they had implemented specific IPC measures after start of the project, with antibiotic stewardship being the most common. Two participants reported that they had initiated leadership walkrounds to highlight the importance of IPC.

### Pre-intervention statements from CMOs regarding their role in IPC

During the kick-off meeting, several CMOs stated that they had thus far underestimated both the scope of IPC measures needed and their role in the management of IPC organization. One CMO said: “*As a medical director, I bear the responsibility in many areas of my hospital, thus IPC has been neglected*”. This was repeated by a CMO of a large non-university hospital: “*Since many of us do our CMO job in addition to being the head of a medical or surgery department, we lack the time to deal with all of these IPC issues*”. Another CMO stated: “*Until my appointment as medical director, I didn´t know that I was also responsible for IPC*.” One CMO expressed the opinion that an IPC training program should be mandatory not only for CMOs but also for chief executive officers (CEO) in order to emphasize the importance of the topic: “*This may convince our CEO that it is necessary to support IPC-projects in my hospitals financially.*” It turned out that the majority of CMOs would appreciate having a practical handbook covering important IPC issues. One participant said: “*I do not have a structured IPC guide that I can work with as medical director.*”

### Secondary endpoints

The pre-intervention pooled median AHR consumption was higher in the intervention group (38.6 ml/PD; (IQR 33.6; 45) than in the control group (33.4 ml/PD; IQR 28.3; 40.8) and there was a post-intervention increase in both groups (Table 2).

**Table 2:** Alcoholic hand-rub consumption and *Clostridioides* (C.) *difficile* cases in intervention hospitals (*n* = 30) vs. control hospitals (*n* = 330) before, during and after intervention, 2017–2019

	**Intervention group (*** n* =** 30)**			**Control group (*n* = 330)**		
	**2017** **(*n* = 23)**	**2018** **(*n* = 24)**	**2019** **(*n* = 19)**	**2017** **(*n* = 218)**	**2018** **(*n* = 217)**	**2019** **(*n* = 207)**
**Alcoholic hand-rub consumption in ml/patient day**						
Mean^a^	40.75	42.49	45.43	36.62	36.85	38
**Median** [IQR]	38.58 [33.59; 44.99]	40.23 [34.00; 51.60]	41.87 [35.01; 56.58]	33.43 [28.29; 40.80]	35.52 [29.47; 42.06]	35.75 [31.60; 43.24]
***C. difficile* cases**						
**Total *C. difficile* cases (cases/ 100 patients)**^b^						
Mean^a^	0.43	0.47	0.33	0.57	0.51	0.4
Median [IQR]	0.30 [0.24; 0.41]	0.30 [0.21; 0.43]	0.24 [0.18; 0.36]	0.38 [0.26; 0.58]	0.35 [0.22; 0.53]	0.29 [0.18; 0.40]
**Incidence density hospital-associated *C. difficile* cases (hospital-associated cases /1000 patient days)**						
Mean^a^	0.32	0.32	0.23	0.38	0.34	0.26
Median [IQR]	0.22 [0,17; 0,33]	0.21 [0.18; 0.33]	0.19 [0.15; 0.26]	0.32 [0.20; 0.48]	0.29 [0.16; 0.45]	0.22 [0.11; 0.33]

*IQR* interquartile Range

^a^ Arithmetic mean

^b^ Hospital-associated and community-associated *C. difficile* cases

**Fig. 1:**
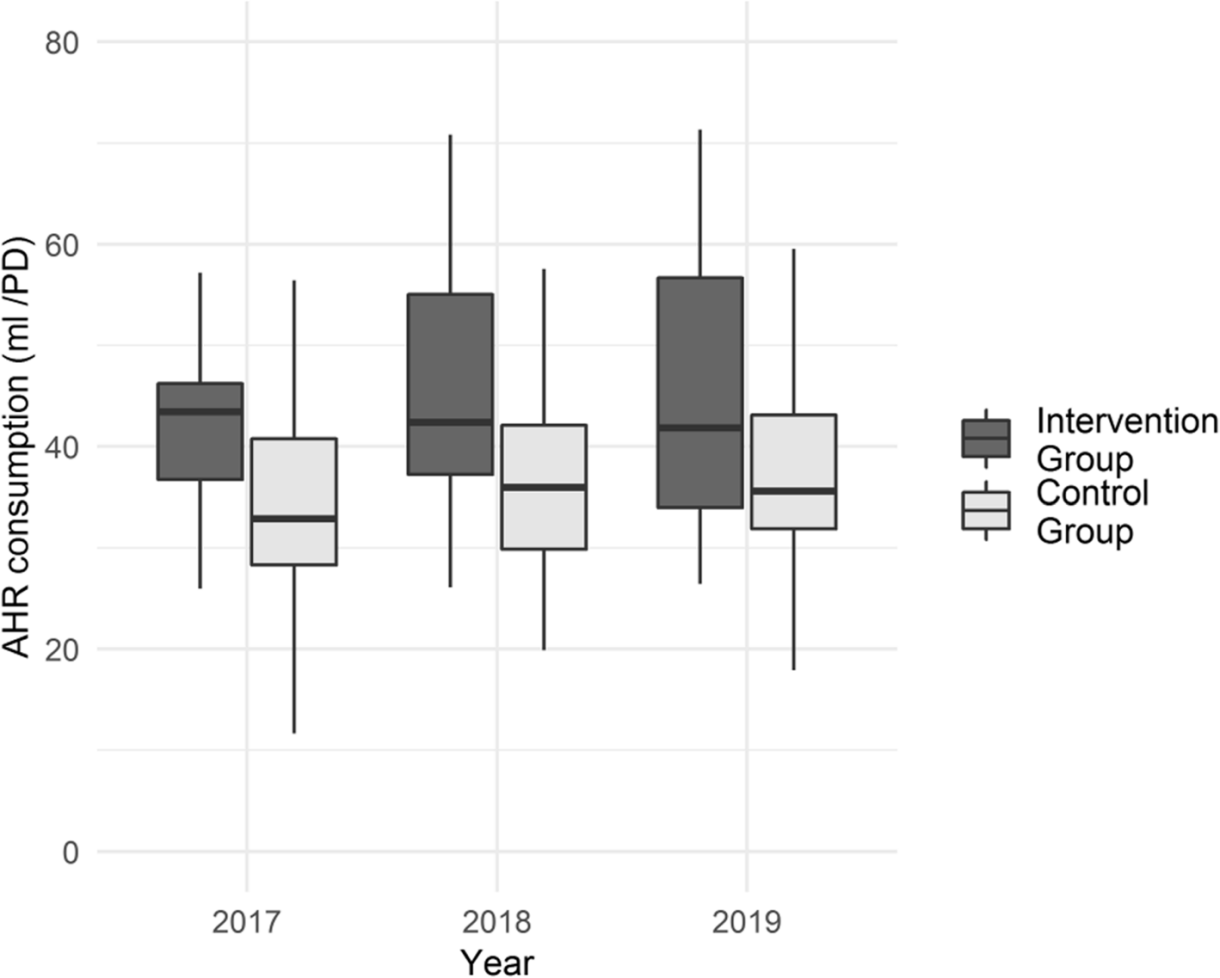
Median consumption of alcohol hand rub in 18 hospitals of the core group* *Only hospitals that recorded data continuously during the years 2017–2019

The analysis of the 18 hospitals of the core group (continuous recording over 3 years) also showed higher baseline AHR consumption for the intervention group (43.4 ml/PD; IQR 36.7; 46.2) than for the control group (33.1 ml/PD; IQR 28.4; 40.9), and an increase in the control group but not in the intervention group. However, between 2017 and 2019, median AHR consumption of the control group increased significantly to 35.6 ml/PD (IQR 31.9; 43.2) but it was still below the baseline value for the intervention group (Fig. 1, Table 3).

**Table 3:** Alcoholic hand-rub consumption and *Clostridioides* (C.) difficile cases in hospitals of the core group** in 2017–2019

	**Core group**^**a**^** Intervention group (*n* = 18)**				**Core group**^**a**^** Control group (*n* = 139)**			
	**2017**	**2018**	**2019**	***p*-value**^**b**^	**2017**	**2018**	**2019**	***p*-value**^**b**^
**Alcoholic hand-rub consumption in ml/patient day**								
Mean^c^	42.75	45.05	45.48		35.4	37.58	37.8	
Median [IQR]	43.43 (36.71; 46.20)	42.41 (37.25; 55.05)	41.86 (33.98; 56.67)	0.1084	33.09 (28.41; 40.86)	36.08 (29.90; 43.09)	35.61 [31.92, 43.17]	< 0.0001
***C. difficile cases***								
**Total* C. difficile cases***^**d**^ (cases/ 100 patients)*								
Mean^c^	0.44	0.46	0.33		0.56	0.5	0.4	
Median [IQR]	0.30 [0.24; 0.41]	0.26 [0.21; 0.39]	0.24 [0.18; 0.36]	0.0043	0.38 [0.26; 0.57]	0.34 [0.22; 0.52]	0.29 [0.18; 0.40]	< 0.0001
**Incidence density hospital-associated* C. difficile* cases (hospital-associated cases /1000 patient days)**								
Mean^c^	0.33	0.3	0.23		0.38	0.33	0.26	
Median [IQR]	0.21 [0.15; 0.35]	0.21 [0.17; 0.28]	0.19 [0.15; 0.26]	0.034	0.32 [0.19; 0.47]	0.28 [0.16; 0.44]	0.22 [0.11; 0.33]	< 0.0001

*IQR* interquartile range

^a^ Group of hospitals that recorded data continuously during the years 2017-2019

^b^ Wilcoxon Rangsummen Test, comparison of 2017 with 2019

^c^Arithmetic mean

^d^Hospital-associated and community-associated *C. difficile* cases

**Fig. 2:**
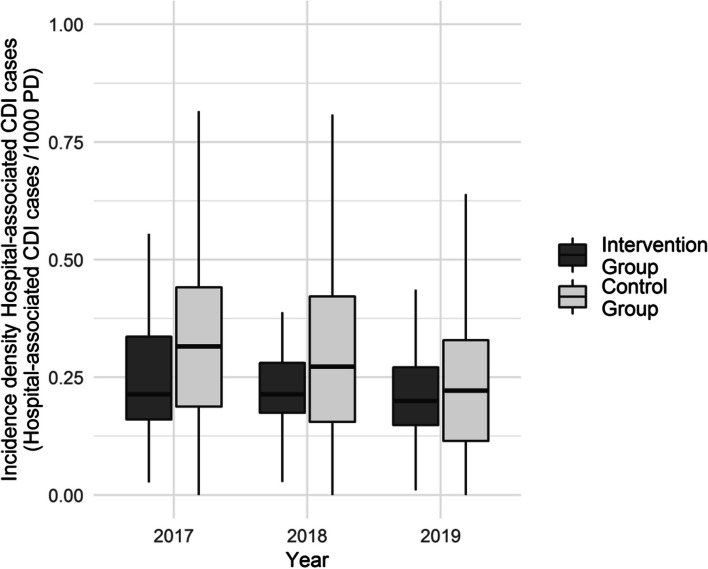
Median incidence density of hospital-associated CDAD in 18 hospitals of the core group* *Only hospitals that recorded data continuously during the years 2017–2019

Regarding *C. difficile*, there was a statistically significant decrease in median hospital-associated CDI cases from 0.32 (IQR 0.19; 0.47) to 0.22 (IQR 0.11; 0.33) CDI cases/1000 PD identified in the control group in the core group analysis (Fig. 2, Table 3) but not in the intervention group, whose baseline rate in 2017 was already lower than the control group’s rate in 2019.

## Discussion

Our study shows that it is worthwhile to offer a multimodal training program among interested CMOs and that training adapted to this target group can provide support in IPC relevant aspects in the respective hospitals.

Our target group were CMOs rather than CEOs or directors of nursing staff since the CMO has primary responsibility for all medical issues including IPC in German hospitals. In a recent survey of German hospital managers, almost all medical managers reported being responsible for the management of hygiene in their institutions, substantially more than nursing or administrative managers [23]. This runs somewhat counter to the recommendation of Brannigan et al., which states that IPC should be a concern of the entire management of a hospital [15]. A quote from a participating CMO confirmed their recommendation regarding the limitations a CMO is under in terms of financial resources for IPC.

However, discussions with the participants revealed that some CMOs were not fully aware of their legal responsibilities for IPC measures or that IPC tasks are often delegated to hospitals’ IPC specialists. Delegating this authorization to IPC specialists implies potential deficits in the implementation of IPC measures since IPC affects primarily interdepartmental and multidisciplinary systems and IPC specialists do not have the competencies to oversee and influence these systems [16].

One benefit of the program is that it makes CMOs aware of their formal responsibilities with the result that they are then able to carry those responsibilities out. In addition it is advantageous for a CMO to possess knowledge about hygiene. That enables them to effectively communicate with their IPC teams on matters concerning hygiene in the hospital [8].

To make these substantial changes, CMOs must first understand their role and full responsibilities as hospital leaders. Since the job of a manager is very time-consuming and provides little opportunity for serious thought about IPC measures, our multimodal approach, consisting of single presence meetings and e-learning tools, appears to be a worthwhile approach.

As of yet, no specific IPC educational tools exist for hospital managers. Certainly, in this regard, CMOs need to be trained in less detail than IPC professionals or IPC link personnel, for whom various training courses are available in Germany, e.g., for IPC link physicians [24]. As concerns management-specific IPC knowledge, we focused on the general requirements for effective IPC programs [5, 21] and on selected national recommendations and legal requirements [24–26]. Training material based on these aspects of IPC rather than on general management tools was influenced by studying organizers’ work experience and can therefore be considered as a particularly *practical* approach to training hospital management. Emphasis was put on organizational aspects, interaction with IPC stakeholders, indicators for monitoring IPC implementation at the hospital-level, including the identification of possible gaps in IPC, and the leadership role of managers’ in implementation processes.

Response to our invitation to participate was not as high as initially expected. Reasons for this are hard to determine, perhaps mangers’ lack of knowledge about their formal responsibility for supporting the design of the IPC activities in the hospital and of the alternatives available was decisive here.

The 30 CMOs who participated in our study represented all three levels of care of acute care hospitals in Germany. The majority of the participants worked in primary care acute care hospitals. This large group also corresponded to the distribution in the control group. Variations in type of care and hospital size did not influence the intense, interactive discussion during the kick-off meeting, indicating that general aspects of IPC at the hospital level are more or less the same for CMOs in hospitals of different types and sizes.

Not all participants registered for the online training. To what extent the knowledge imparted at the kick off meeting was sufficient to account for possible changes in the corresponding hospitals cannot be assessed. The entire educational intervention was based exclusively on voluntary participation and did not contain a final examination of the knowledge acquired.

Interestingly, participants asked whether a script could be created as another form of knowledge transfer. Even though the use of the online training courses was very flexible the script form seemed to appeal even more to a large number of the participants.

In total, participants considered information about management’s IPC tasks useful. This positive assessment—that knowledge of IPC had increased—was also reflected in the replies to the final survey of the changes in CMOs’ IPC management. They reported that IPC measures were implemented and communication regarding IPC had been intensified. Head physicians were the occupational group which experienced the greatest improvement in communication on IPC issues with the CMOs. This is a valuable result since chief physicians also play an important role in the implementation of IPC measures. Communication across occupational groups also improved, a valuable contribution to safety culture.

However, although several changes were implemented by the participants, we were not able to show a statistically significant effect on our secondary outcomes. In fact, AHR consumption and numbers of CDI cases remained stable in the intervention group, we observed a statistically significant increase in AHR consumption and a statistically significant decrease in the CDI cases in control group hospitals. These findings might be explained by the following: At start of our study, AHR consumption rates were significantly higher statistically and CDI numbers lower in interventional hospitals than in control hospitals. This might indicate selection bias, with intervention hospitals that already had higher compliance rates with recommended IPC measures at the start of our study and where further improvements were therefore hard to achieve. It has been shown that hospitals with more obvious gaps in IPC benefit more from implementation of corresponding IPC measures. In Germany, for example, AHR consumption has increased considerably during the last decade, and hospitals which started with a low AHR consumption achieved the largest increase [27]. Secondary outcomes were measured during the intervention year and shortly afterwards, which could indicate that the time span was too short to achieve statistically significant results. This might also be influenced by the small sample size of our intervention group, since we identified a small but statistically insignificant decrease in total *C. difficile* infections and in the incidence density of hospital-associated *C. difficile* cases. However, whether the hospital-based endpoints selected are suitable for measuring the effects of the implementation of an education program for CMOs also needs to be discussed. Sneddon et al. have also reported that measuring the impact of training programs remains challenging [28].

Our study has the following additional limitations: Because participation of CMOs was voluntary, the results are not representative of all German acute care hospitals. But even a systematic and randomized sampling would not have been representative in terms with the low response rate of 8%. However, the low response rate can also be seen as a result of our study, that the vast majority of CMOs not (yet) knowing the important significance of their potentially active role in IPC or not giving this role a high priority in terms of training. The fact that the hospitals participating in the intervention had a better baseline level of AHR consumption and CDI rates, representing a specific group of hospitals with a potentially higher interest in sufficient IPC, emphasizes the selection bias in our study. The selected hospitals of the core group may also be associated with selection bias.

A further limitation of this study is that participants reported changes in IPC management during the intervention which were not confirmed externally. The change in the study design as a result of the lower number of participants must also be mentioned in this context.

Despite these limitations, we conclude that education of interested CMOs by means of a multimodal IPC program is worthwhile and that such a program can lead to improvements in individual hospitals. However, due to the small sample size, the short time frame of the study, and selection bias issues, it has yet to be shown whether such a program will actually affect hard IPC-endpoints such as AHR consumption or decreases rates of infectious diseases such as CDI. At the present time, it is planned to continue the study and to strengthen additional CMOs in their knowledge of IPC in order to eventually reach the goal of minimizing infection and transmission risks in health care. The extent to which training can also be extended to non-medical hospital management needs to be further examined.

### Supplementary Information

**Additional file 1.** LEAD-IC short evaluation questionnaire.

**Additional file 2. **Study Protocol.

**Additional file 3:** **Supplement Table 1.**

## Data Availability

The datasets used and/or analysed during the current study available from the corresponding author on reasonable request.
