# Supplementary material for: Strengthening the role of hospital leadership in infection control (LEAD-IC) – a multimodal educational intervention in German acute care hospitals
Source: BMC Med Educ. 2023 Oct 11;23:758. doi: 10.1186/s12909-023-04709-z (PMC10568750; doi:10.1186/s12909-023-04709-z)
Supplement: Supplementary file 1 — Additional file 1. LEAD-IC short evaluation questionnaire. [file 12909_2023_4709_MOESM1_ESM.pdf]

**LEAD-IC short evaluation questionnaire**

*Dear LEAD-IC-Participant,*

*In the following 5 questions we would like to evaluate whether you have implemented changes in infection prevention and control (IPC) measures in your hospital in the last few months. Please feel free to provide keywords in the free text fields.*

**During the last few months…**

1. **… I have changed cooperation/interaction regarding IPC measures with the following individuals/institutions (multiple answers possible):**

   **O Chief physicians**

   If yes, in what form? (free text):

   ______________________________________________________________________________
   ______________________________________________________________________________
   ______________________________________________________________________________

   **O Administration**

   If yes, in what form? (free text):

   ______________________________________________________________________________
   ______________________________________________________________________________
   ______________________________________________________________________________

   **O Chief executive officer**

   If yes, in what form? (free text):

   ______________________________________________________________________________
   ______________________________________________________________________________
   ______________________________________________________________________________

   **O Nursing management**

   If yes, in what form? (free text):

   ______________________________________________________________________________
   ______________________________________________________________________________
   ______________________________________________________________________________

   **O Local health authorities**

   If yes, in what form? (free text):

   ______________________________________________________________________________
   ______________________________________________________________________________
   ______________________________________________________________________________

**2. … I have made changes in cooperation/interaction with the IPC team:**

O Yes

If yes, in what form? (free text):

___________________________________________________________________________
___________________________________________________________________________
___________________________________________________________________________

O No, I have not made any changes because cooperation/interaction with the IPC team is already very good.

O No, I have not made any changes.

3. **… I have made changes in the planning or implementation of the IPC committee meetings:**

O Yes

If yes, in what form? (free text):

___________________________________________________________________________
___________________________________________________________________________
___________________________________________________________________________

O No, I have not made any changes because IPC committee meetings procedures are already very good.

O No, I have not made any changes.

**4. … I have arranged for the content of the following IPC measures to be revised/adapted:**

**O Screening for multidrug-resistant organisms (MDRO)**

If yes, in what form? (free text):

___________________________________________________________________________
___________________________________________________________________________
___________________________________________________________________________

**O Isolation of patients with MDRO**

If yes, in what form? (free text):

___________________________________________________________________________
___________________________________________________________________________
___________________________________________________________________________

**O Outbreak management**

If yes, in what form? (free text):

______________________________________________________________________
______________________________________________________________________
______________________________________________________________________

**O Internal IPC guidelines**

If yes, in what form? (free text):

______________________________________________________________________
______________________________________________________________________
______________________________________________________________________

**O Other**

Free text:

______________________________________________________________________
______________________________________________________________________
______________________________________________________________________

**5. … I have implemented or started implementing the following concrete IPC measures:**

**O WalkRounds**

If yes, in what form? (free text):

______________________________________________________________________
______________________________________________________________________
______________________________________________________________________

**O Antibiotic Stewardship**

If yes, in what form? (free text):

______________________________________________________________________
______________________________________________________________________
______________________________________________________________________

**O Other**

Free text:

______________________________________________________________________
______________________________________________________________________
______________________________________________________________________
