# Supplementary material for: Strengthening the role of hospital leadership in infection control (LEAD-IC) – a multimodal educational intervention in German acute care hospitals
Source: BMC Med Educ. 2023 Oct 11;23:758. doi: 10.1186/s12909-023-04709-z (PMC10568750; doi:10.1186/s12909-023-04709-z)
Supplement: Supplementary file 2 — Additional file 2. Study Protocol. [file 12909_2023_4709_MOESM2_ESM.pdf]

**Study Protocol**

Leadership in Infection Control

Die Rolle der Krankenhausleitung in der Infektionsprävention

**(LEAD-IC)**

**Document history**

| Created/modified by | Date | Version |
| --- | --- | --- |
| C. Remschmidt | 14.06.17 | V01 |
| | | |

**Background**

Effective measures which are known to prevent nosocomial infections and reduce antibiotic resistance are not always fully implemented in German hospitals. Since 2011 the Infection Prevention Act (Infektionsschutzgesetz, IfSG) and state hygiene ordinances have made hospital management responsible for compliance with infection prevention measures. This task is usually assigned by the management of a hospital to its chief medical officer (CMO). In most cases, however, these medical officers do not have the appropriate professional background to perform this task. Expertise in the field of infection prevention and control (IPC) is found mainly in the hospital hygienist and hygiene specialists. However, when it comes to implementing prevention measures—measures that as yet have not been successfully implemented—these staff members often do not have sufficient authority or influence to carry out the necessary changes. The fact that support from hospital management has a positive effect on compliance with prevention measures has been demonstrated in several studies (Brannignan et al, Frankel et al, Siant et al, Sinkowitz-Cochran et al). Within the framework of support, barriers can be identified and reduced and employees motivated to implement prevention measures. Active support on the part of management (leadership) is thus an important contribution to the culture of safety in hospitals.

Up to now, the cooperation of the above-mentioned individuals, which is urgently needed for infection prevention, has been concentrated in most hospitals on the hygiene commission meetings required by the IfSG and which have to be held regularly. These commission meetings are an important forum for analysing the current status of infection prevention, identifying priorities for further work, and discussing the introduction of further measures. However, the interaction between the CMO and a hospital's IPC staff at IPC commission meetings is usually not adequate in the long term for successfully implementing the measures necessary.

In a 2014 survey of 534 KISS hospitals, 79% of the senior IPC staff reported that the hospital management was involved in the IPC commission. However, concrete objectives with regard to specific IPC-relevant process indicators were only set in 13% of the hospitals. Only 47% of the senior IPC staff felt they were actively supported by hospital management in

implementing IPC measures, and in only 25% of the hospitals did the hospital management offer "rounds" (so-called "WalkRounds") (Hansen et al., Bundesgesundheitsblatt 2016). Within the framework of standardized, multicentre training courses on sepsis prevention in intensive care units (ISEP, Hansen et al.) and neonatal intensive care units (NEO-ISEP, Salm et al., both within the framework of the Hospital Infection Surveillance System (KISS)) and the "Clean Hands Campaign," it has been shown that there is great interest to do more in the field of infection prevention in many hospitals. In the ISEP and NEO-ISEP projects, a significant reduction in bloodstream infections was achieved within the framework of such training materials.

**Objective**

Building on these experiences, the project "LEAD-IC" ("Leadership in Infection Control" or "The Role of Hospital Management in Infection Prevention") is to be developed. The LEAD-IC project is intended to support hospital management (usually the CMOs) with a structured approach that will better prepare them for their tasks, as specified by the IfSG, and to improve the implementation of IPC measures through the competence and commitment they get from the project. This is a first in Germany.

Important starting points for effective design of infection prevention in hospitals are to be conveyed within the framework of a structured multimodal training program. This is also intended to establish synergies between the hospital management and the hygiene team of a hospital for overcoming barriers and to improve IPC.

**Primary study objective**

Can structured multimodal training of hospital management reduce the incidence of bloodstream infections (BSI) in hospitals?

**Secondary study objectives**

Can a change in the following indicators be achieved through structured, multimodal training of hospital management?

i.) A reduction in the incidence of bloodstream infections by multidrug-resistant organisms (MDRO) and/or
ii.) a reduction in the incidence of nosocomial *Clostridium difficile* infections (CDI)
iii.) an increase in the hospital-wide consumption of alcohol-based hand disinfectant.

## Methods

### Study design and inclusion criteria

LEAD-IC will be conducted as a prospective, cluster-randomised, controlled intervention study of stepped-wedge design. Eligible participatants are the EDs or their deputies of those hospitals in Germany that have been participating in Hand-KISS (Surveillance of Hand Disinfectant Use (HDMV)), MRSA-KISS and CDAD-KISS (Surveillance of *Clostridium difficile*-associated diarrhea) modules of the National Reference Centre for Surveillance of Nosocomial Infections (NRZ) since 2016 and for at least 18 months (n=402, as of 04/2017). These hospitals are to be randomly divided into 2 groups: Group 1 hospitals will have the opportunity to participate in the intervention during the first intervention period (year 2018), while Group 2 hospitals will have the opportunity to participate in the intervention during the second intervention period (year 2019). The control group will be (i) the respective intervention group before the start of the intervention, (ii) in 2018, intervention group 2 will serve as a control for intervention group 1. Before the intervention and after the end of the intervention period, IPC structures of the hospital will be surveyed by means of a structured questionnaire.

### Recruitment

The CMOs of all 402 hospitals will be contacted by post and invited to participate in the study. Doctors (or their deputies) who agree in writing to participate will be invited to an introductory event and later will be given training online over a 5-6 week period (see below).

### Randomization and blinding

All participants who have given their written consent to participate will be randomly assigned to one of the two intervention groups. A later change of this assignment (e.g. due to scheduling difficulties of the participants) will not be possible. Participants will be instructed in the introductory session not to inform their co-workers about the study. This is to avoid the intervention (training of the doctors) being influenced by knowledge of the study by other hospital staff. Blinding of the participants with regard to the endpoints is not planned.

**Endpoints**

**Primary endpoint:** The rate of bloodstream infections per hospital per year, measured as number (n) of all positive blood cultures (BC) per 1000 patient days/ number (n) of all BK per 1000 patient days. Since data on blood cultures taken is not routinely collected for the entire hospital in the KISS system, the hospitals must request this data from the department responsible for the data or the responsible microbiological laboratory and transmit it to the study center. The methodological approach of using BC at hospital level as an endpoint has already been carried out in other studies (Rock et al, 2016; Leekha et al, 2013). Analogous to the study by Rock et al., the collection of data on the primary endpoint should be uncomplicated and only the absolute values of BC or positive BC per year should be requested. The possibility of multiple collections from a single patient or possible contamination of the BCs with coagulase-negative staphylococci should not be taken into account primarily as it is assumed that this figure will not be influenced by the intervention. For the **secondary endpoints**: In order to calculate the rate of bloodstream infections by multi-resistant pathogens (MRE) per hospital per year (number (n) of all positive blood cultures (BC) per 1000 patient days/ number (n) of all BC with evidence of MRE per 1000 patient days), the pathogen type will also be recorded when a BC is positive and transmitted to the study center. The determination of resistance will be carried out by the respective hospital laboratory, whereby the participating hospitals must specify the methodology used for testing resistance (EUCAST, DIN 58940 or CLSI (Clinical & Laboratory Standards Institute)). The other **secondary endpoints** (incidence of nosocomial *Clostridium difficile* infections and the consumption of alcohol-based hand disinfectant per hospital per year) are recorded directly via KISS.

**Study period**

The intervention will take place over 12 months in 2018 (group 1) and over 12 months 2019 (group 2) and will begin with an introductory event in the first quarter. Data collection will take place at the beginning of the study and at the end of the intervention phase. Online training will start for both groups in the first quarter of the intervention and run for a period of 5-6 weeks. In the 2nd quarter, 2 reminders will be sent to the participants.

## Work plan and timetable of the LEAD-IC project

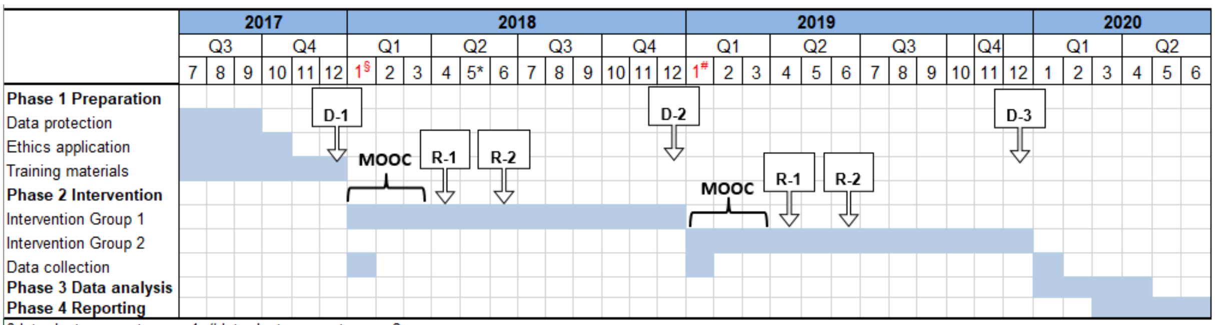


§ Introductory event group 1; # Introductory event group 2
D1-D3: Timing of data collection at hospital level
R1, R2: R1, R2: Reminder to participate in the online trainings
MOOC (Massive Open Online Course): Online training over 6-8 weeks in the form of thematically sorted, weekly blocks

## Sample size calculation

The calculation of the sample size is based on an expected decrease of 15% in the primary endpoint (nosocomial BSI).

Based on studies in R-net (network of the DZIF), a median BSI incidence of 0.5 per 100 patients can be assumed. Assuming an alpha error of 0.05, a power of 80%, an expected reduction effect by the intervention of 15%, and a design effect (which was estimated on the basis of KISS ICU data on catheter-associated bloodstream infections with an intra-cluster correlation of 0.13), the sample size will be approx. 130,200 patients per group.

On average, approx. 15,000 patients per hospital and per year are to be expected. Accordingly, at least nine hospitals per group are to be included. Taking into account possible "dropouts," it is planned to include 15 hospitals per group (calculated by Christin Schröder on 25.07.17).

## Intervention (multimodal training of hospital management)

The intervention starts in both groups with a one-day introductory event at the beginning of the intervention period. Here, an introduction to the study will be presented and preliminary knowledge provided about the organization of the IPC team and procedures in the event of outbreaks (topics 1 & 2, see below). During the next 5-6 weeks, the participants will be trained online in five additional topic complexes to be structured as follows (topics 3-7):

| Nr. | Topic | Mode |
|---|---|---|
| 1 | • What is the optimal organization of the hygiene team and the liaison staff to the wards (doctors responsible for hygiene, nursing staff responsible for hygiene) based on the type and size of the hospital?<br>• What aspects must be considered when outsourcing important services related to infection prevention (microbiological laboratory, central sterilization, cleaning, etc.)?<br>• What must be considered when establishing antibiotic stewardship (ABS) concepts? | Face-to-face meeting |
| 2 | • How do you organise good crisis management during outbreaks? | Face-to-face meeting |
| 3 | • How is a good overview of infection rates, resistance rates, and the implementation of processes obtained, based on conditions in each hospital?<br>• What is the best way to support the hygiene staff in terms of data management? | Online (Week 1) |
| 4 | • How should hygiene committee meetings be organised so that they improve the implementation of prevention measures?<br>• Is it useful to define hospital-wide prevention targets, and which targets are realistic? | Online (Week 2) |
| 5 | • From a hospital hygiene point of view, what should one be aware of in hospital management "WalkRounds"? | Online (Week 3) |
| 6 | • Which legal aspects have to be taken into account? How can cooperation with health authorities be optimized? | Online (Week 4) |
| 7 | • What are sensible and pointless measures, and how can "old habits" be broken? | Online (Week 5) |

**Implementation of the online training**

The online training courses will take place in the form of MOOCs (Massive Open Online Courses) in 5 weekly blocks. Participants must register one time on the corresponding internet platform with an email address and a password and will then receive an email at the

beginning of each week reminding them to participate. The content of MOOCs is conveyed primarily in the form of video sequences. Learning materials can also be downloaded from the platform, and participants can exchange information on forums. After each weekly block, there will be a multiple-choice test that checks individual knowledge. The results of the multiple-choice test will be reported back to the participants individually; the study center will receive the results anonymously. The weekly blocks consist of 3-5 video sequences, each 5-10 minutes long. The total weekly training time should not exceed 60 minutes. A hotline to the study center, which is active at pre-determined times, will give participants an opportunity to ask questions when uncertainties arise.

**Data protection and ethics**

No personal data of patients will be collected or transmitted. A data protection vote and a positive vote by the ethics committee have been obtained for the anonymized collection and evaluation of the cumulative KISS data. For the current project, a data protection application and an ethics application will be submitted to the Charité, and the study will begin only after a positive vote. The data of the hospitals and the responsible hospital management will be evaluated pseudonymously, aggregated in the study center, and published. The heads of the participating hospitals must give their consent for participation in the study.

**Cooperations**

Within the framework of the study, cooperation is to be entered into with the following institutions:

- German Hospital Association (DKG)

Support in the recruitment of participating hospitals

- Association of Hospital Directors in Germany (Verband der Krankenhausdirektoren Deutschlands eV), requested

Support in recruiting the participating hospitals

Support in the design of the training materials with regard to their target group-specific applicability

**Gender aspects**

The implementation of the intervention will also analyze whether there are differences between male and female hospital managers.

**Benefit and use of the results/ sustainability**

If the intervention at hospital level leads to a reduction in the incidence of nosocomial infections, the number of nosocomial infections in general can be reduced in the future, with a concommitant reduction in treatment duration, complications, and costs.

**Possible risk factors**

<u>Participation rate</u>

According to the German Hospital Association, there is great interest among many hospital managers/medical directors in additional training in this area. The Association of Hospital Directors are interested as well. Therefore, the risk of possible under-recruitment of interested hospital directors is considered to be very low.

<u>MOOCs</u>

In case of unexpected, non-recoverable technical problems with the MOOCs, classical training concepts (PowerPoint presentations) will, if necessary, be used.

**Projektmanagement**

Responsibility/ Role

| Name | Institut | Role |
|---|---|---|
| Dr. Sonja Hansen | Institut für Hygiene und Umweltmedizin, Charité – Universitätsmedizin Berlin | Project management |
| Prof. Dr. Petra Gastmeier | Institut für Hygiene und Umweltmedizin, Charité – Universitätsmedizin Berlin | Project management |
| Dr. Cornelius Remschmidt | Institut für Hygiene und Umweltmedizin, Charité – Universitätsmedizin Berlin | Study physician |
| Dr. Frank Schwab | Institut für Hygiene und Umweltmedizin, Charité – Universitätsmedizin Berlin | Supervision Statistical analysis |
| Dr. Michael Behnke | Institut für Hygiene und Umweltmedizin, Charité – Universitätsmedizin Berlin | Supervision Data management |

**Unterstützende Einrichtungen/ Kooperationspartner**

| Name | Institut | Role |
|---|---|---|
| Herr Georg Baum | Deutschen Krankenhaus-gesellschaft (DKG) | Support in the recruitment of participating hospitals |
| Frau Gabriele Kirchner, angefragt | Verband der Krankenhaus-direktoren Deutschlands e.V. | Support in the recruitment of participating hospitals; Support in the design of the training materials with regard to their target group-specific applicability. |
| | | |

**Literature**


- Brannigan E.T., Murray E., Holmes A. Where does infection control fit into a hospital management structure? J Hosp Infect 2009; 73:392-396.

- Frankel A., Grillo S.P., Baker E.G. et al. Patient Safety Leadership WalkRounds at Partners Healthcare: learning from implementation. Jt Comm J Qual Patient Saf. 2005; 31:423-437.

- Hansen, S., Schwab, F., Gropmann, A. et al. Hygiene und Sicherheitskultur in deutschen Krankenhäusern. Bundesgesundheitsblatt Gesundheitsforschung Gesundheitsschutz, Juni 2016, in press

- Saint S., Kowalski C.P., Banaszak-Holl J. et al. The importance of leadership in preventing healthcare-associated infection: results of a multisite qualitative study. Infect Control Hosp Epidemiol 2010; 31:901-907.

- Sinkowitz-Cochran R.L., Burkitt K.H., Cuerdon T. et al. The association between organizational culture and knowledge, attitudes, and practices in a multicenter Veterans Affairs quality improvement initiative to prevent methicillin-resistant Staphylococcus aureus. Am J Infect Control 2012; 40:138-143.

- Rock C., Thom K.A., Harris A.D. et al. A Multicenter Longitudinal Study of Hospital-Onset Bacteremia: Time for a New Quality Outcome Measure? Infect Control Hosp Epidemiol 2016; 37:143-148.

- Zingg W., Holmes A., Dettenkofer M. et al. Hospital organization, management, and structure for prevention of health-care-associated infection: a systematic review and expert consensus. Lancet Infect Dis. 2014; 15: 212 – 224.
