# Supplementary material for: Strengthening the role of hospital leadership in infection control (LEAD-IC) – a multimodal educational intervention in German acute care hospitals
Source: BMC Med Educ. 2023 Oct 11;23:758. doi: 10.1186/s12909-023-04709-z (PMC10568750; doi:10.1186/s12909-023-04709-z)
Supplement: Supplementary file 3 — Additional file 3: Supplement Table 1. [file 12909_2023_4709_MOESM3_ESM.docx]

**Supplement Table 1: Baseline characteristics of the participating hospitals (n=30) and the control group (n=330)**

| Variable | Intervention group (n=30) | Control  group (n=330) | p-value* |
| --- | --- | --- | --- |
| Number of beds |  |  |  |
| - Median (interquartile range) | 300 (178; 493) | 357 (25; 595) | p=0. 04 |
| Level of care |  |  |  |
| - Tertiary care hospitals (including university hospitals, n (%) | 6 (20) | 27 (8) | <0.01 |
| - Secondary care hospitals, n (%) | 5 (17) | 59 (18) |  |
| - Primary care hospitals, n (%) | 19 (63) | 237 (72) |  |
| - No information | 0 | 7 (2) |  |

*Chi-square test
